# Participants' Experiences and Satisfaction With Sun Protection Factor (SPF) 100 Sunscreen in Actual Use Conditions

**DOI:** 10.7759/cureus.61212

**Published:** 2024-05-28

**Authors:** Maria Naseer, Nazia Asad, Armeela Javaid, Ramla Moughal, Hadia Akram, Shafia Mudassir, Muhammad Iqbal Asif, Neeta Maheshwary, Arjumand Ahmed

**Affiliations:** 1 Department of Dermatology, Shaheed Benazirabad Peoples University of Medical & Health Sciences for Women, Nawabshah, PAK; 2 Department of Dermatology, Jinnah Postgraduate Medical Center (JPMC), Karachi, PAK; 3 Department of Dermatology, La Chirurgie, Islamabad, PAK; 4 Department of Dermatology, Anam Hospital, Karachi, PAK; 5 Department of Dermatology, Silk Skin Clinic, Rawalpindi, PAK; 6 Department of Medicine, Karachi Medical and Dental College, Karachi, PAK; 7 Department of Medical Affairs, Helix Pharma Pvt. Ltd., Karachi, PAK

**Keywords:** sunscreen, satisfaction, sun protection factor, sunburn, spf100

## Abstract

Background and objective

Exposure to sunlight's ultraviolet (UV) radiation poses various health risks, including sunburn, skin damage, and heightened skin cancer risk. Sunblock usage has surged due to widespread advertising campaigns. Individuals spending time outdoors should employ protective measures like wearing hats, applying sunblock with a high sun protection factor (SPF), covering exposed skin, and seeking shade to mitigate UV exposure's harmful effects. This study's objective is to assess participants’ experiences and satisfaction with SPF 100 sunscreen in actual use conditions.

Methodology

This study employed a prospective, single-center design involving 100 participants aged 18 to 70 years. Eligible individuals had Fitzpatrick skin types I-III and were engaged in outdoor activities, excluding those with certain medical conditions or medication use. Each participant received sunscreen tubes (Solero SPF 100, Helix Pharma Pvt. Ltd., Karachi, Pakistan), and clinical evaluations were conducted on the day before and after and day 22 visits, with sunblock application and UV-induced erythema assessments performed.

Results

Our study enrolled participants with a mean age of 25.6 ± 7.1 years, ranging from 15 to 55 years, with females comprising 84% (84) of the sample. Results revealed widespread satisfaction and acceptance of SPF 100 sunscreen, without any reported adverse reactions. A significant majority expressed their willingness to purchase and recommend the sunscreen to others. Furthermore, the majority of healthcare providers expressed satisfaction with prescribing this sunscreen.

Conclusions

In conclusion, SPF 100 sunscreen demonstrated excellent tolerability and acceptability among participants, suggesting its potential utility in both personal sun protection routines and clinical settings.

## Introduction

The sun emits a range of radiation, including infrared (IR), visible light, and ultraviolet (UV) radiation, all falling under the electromagnetic spectrum spanning from 100 nm to 1 mm [1]. Among UV radiation, there are three subtypes: UVA, UVB, and UVC. UVA constitutes about 95% of the UV rays reaching Earth, while UVB makes up approximately 5%. Both UVA and UVB have the potential to damage DNA, with UVA primarily causing tanning and skin aging, and UVB penetrating deeper into the skin, leading to sunburn and other conditions [2-3]. Prolonged exposure to UV radiation can result in severe complications such as eye disorders and skin cancer [4]. Additionally, IR and visible light also negatively impact the skin, causing alterations in the outermost layer and degradation of the deeper skin structure [5].

Preventing sun damage requires taking appropriate measures. According to the World Health Organization (WHO), wearing protective clothing, sunglasses, and hats alongside applying sunblock to exposed skin areas provides the best defense against UV radiation [6]. Daily use of sunblock has been shown to reduce the incidence of skin cancer and premature skin aging [7]. Sunblocks work by utilizing active ingredients that scatter, absorb, or reflect UV rays to protect the skin [8].

The active ingredients of sunblocks can be organic or inorganic, and their use is regulated by various agencies worldwide. For instance, the US FDA has approved 16 sunblock active ingredients, while Health Canada, the European Commission, the Australian Therapeutic Goods Administration, and China's NMPA have approved different sets of ingredients [9]. In addition to synthetic compounds, natural molecules are also employed in sunblock formulations due to their photoprotective properties. These include carotenoids, phenolic compounds, vitamins C and E, and flavonoids. Their use underscores the growing interest in harnessing nature-derived ingredients for sun protection [10].

The advancement of nanotechnology has further enhanced the efficacy of sunblock by improving its efficiency, stability, and bioavailability. Nanotechnology enhances the effectiveness and duration of action of these products, offering new avenues for skin care and sun protection [11]. The emergence of nanocosmeceuticals represents a significant development in cosmeceutical products aimed at improving various skin conditions, including sun protection [12].

In recent studies, the sun protection factor (SPF) of sunblock has been highlighted as crucial for shielding against harmful UV radiation, which can lead to sunburn and skin cell damage. High-SPF sunblocks are believed to offer superior protection, particularly in real-world scenarios where users may apply them at lower densities than in standardized SPF testing methods [13-14].

According to dermatologists surveyed, over 80% believe that high-SPF sunblocks provide an added safety margin [15]. Research by Russak et al. found that an SPF85 sunblock outperformed an SPF50 sunblock in preventing sunburn during high-altitude skiing [16]. Additionally, findings from a recent study by Josha et al. revealed that SPF 100+ sunblock significantly reduced erythema severity after a single exposure, indicating enhanced sunburn protection. Furthermore, sunblock with an appropriate SPF is essential for shielding against UV radiation-induced skin damage. Studies have shown SPF 100+ sunblock to be significantly more protective against sunburn compared to SPF 50+ sunblock, particularly in settings like springtime recreational snow skiing [13-17]. Kohli et al. reported that after cumulative sun exposure, areas protected by SPF 50+ were more frequently assessed as sunburned compared to areas protected by SPF 100+. Mean clinical erythema scores were consistently lower on the SPF 100+ side, both cumulatively and after the first day of exposure, highlighting its superior efficacy in preventing sunburn [18].

The rationale for our study stems from a gap in existing research regarding the effectiveness of very high-SPF sunblocks for protecting against sunburn in real-world conditions over multiple consecutive days of sunlight exposure, specifically within our country. Despite the widespread use of high-SPF sunblocks, there is ongoing debate about the extent of additional protection they offer beyond lower SPF formulations. By doing so, we hope to provide valuable insights into the practical efficacy of these sunblocks and contribute to the ongoing discourse surrounding their use and effectiveness. The objective of this study was to assess the Participant's Experiences and Satisfaction with SPF 100 sunblock (Solero, Helix Pharma Pvt. Ltd., Karachi, Pakistan) in actual use conditions.

## Materials and methods

This prospective, observational, single-center study aimed to assess the participants’ experiences and satisfaction with using SPF 100 sunblock (Solero) to prevent and treat UV radiation's skin-damaging effects. A real-world evidence approach was adopted, with a minimum sample size of 100 study participants aged between 18 and 70 years. The estimated sample size for our study was 90 participants, allowing for a 10% nonresponse rate (*n *= 100). With a statistical power of 90% and a significance level of 0.03, the 95% efficacy of SPF 100+ sunblock in preventing sunburn is based on previous research [17].

The study was conducted at Peoples University of Medical and Health Sciences for Women, Shaheed Benazirabad, Nawabshah, Pakistan (IRB# PUMHSW/SBA/PVC/2023/294, June 6, 2023), and all participants provided written consent. Eligible participants included healthy men and women aged 18 years or older with Fitzpatrick skin types I-III who were engaged in outdoor activities. Exclusion criteria were a history of adverse effects upon sun exposure, current use of photosensitizing medications, or preexisting conditions such as cancer, lupus, or diabetes that could increase risks associated with study participation.

During the baseline visit, participants were evaluated for eligibility criteria, including good general health, capability of understanding the study protocol, and willingness to have the test materials applied. Detailed demographic data were collected, including assessments before application of Solero, after application, and at subsequent follow-up visits (day 22). Any adverse events or unexpected outcomes were closely monitored and reported.

During screening, exposed skin areas were clinically examined, including the face and neck, arms and shoulders, and lower legs and feet. Each participant received sunblock tubes (Solero SPF 100) and a sun exposure diary to record their outdoor time. UV-induced erythema was assessed clinically on day 1 before, day 1 after, and on day 22 by an investigator. Participants exhibiting sunburn (erythema score ≥ 2) were excluded from participation in remaining sun exposure. 

The data collection instrument utilized in our study consisted of a structured questionnaire designed to gather pertinent information regarding participants' perceptions and experiences with SPF 100 sunscreen. This questionnaire underwent rigorous development and validation procedures to ensure reliability and validity. The Cronbach's alpha coefficient for the questionnaire in our study was calculated to be 0.863, indicating a high level of internal consistency among its items. Data collection was conducted through face-to-face interviews administered by trained research personnel. Before the commencement of the study, participants were provided with detailed information about the purpose and procedures involved. Written informed consent was obtained from all participants before their inclusion in the study. During the interviews, participants were asked a series of standardized questions about their satisfaction, acceptance, and willingness to use SPF 100 sunscreen. Additionally, healthcare providers were queried regarding their satisfaction with prescribing sunscreen to patients.

IBM SPSS Statistics for Windows, Version 23.0. (IBM Corp., Armonk, NY) was utilized for both data entry and statistical analysis in this study. For categorical variables, numbers and frequencies were calculated, while means and standard deviations were computed for numerical variables.

## Results

The mean age of the participants was 25.6 ± 7.1 years. The age range varied from a minimum of 15 years to a maximum of 55 years. The majority of participants were female, accounting for 84 (84%) of the sample, while males constituted 16 (16%). All participants had normal skin characteristics, with none presenting with disordered skin. Additionally, no specific diagnoses were provided for any participant.

On day 1 before the application of SPF 100 sunblock, participants' responses regarding sun protection behaviors and preferences were collected. Among the participants, 46 (46%) strongly agreed and 33 (33%) agreed that they experienced flushing or blushing strongly when exposed to sunlight. Additionally, 31 (31%) participants strongly agreed and 33 (33%) agreed that they had sensitive skin to products applied to the face. A significant majority of participants, comprising 48 (48%), strongly agreed, and 42 (42%) agreed that they preferred selecting products well tolerated on their face. The majority of participants, accounting for 72 (72%), reported using sunblock before going into the sun. Concerning SPF preferences, 27 (27%) participants used SPF < 30, 26 (26%) used SPF 30-50, 29 (29%) used SPF 50+, and 18 (18%) used SPF 100. Moreover, the majority of participants, representing 76 (76%), expressed satisfaction with their current sunblock (Table 1).

**Table 1:** Study participants' responses on day 1 before application.

Questions	Strongly agree	Agree	Disagree	Strongly disagree
1. Do you flush and blush strongly or get are face in the sun?	46 (46%)	33 (33%)	13 (13%)	8 (8%)
2. Do you have sensitive skin to products applied to the face?	31 (31%)	33 (33%)	28 (28%)	8 (8%)
3. Do you prefer to pick out products that are well tolerated on your face?	48 (48%)	42 (42%)	7 (7%)	3 (3%)
4. Do you sunblock before going in the sun? (Yes)	72 (72%)			
5. What sun protection factor do you use most often? (Yes)	<30	30-50	50+	SPF 100
	27 (27%)	26 (26%)	29 (29%)	18 (18%)
6. Are you satisfied with your current sunscreen? (Yes)	76 (76%)			

Upon application of SPF 100 sunblock on day 1, participants provided feedback on their experiences and perceptions of the product. A significant portion, comprising 46 (46%) participants, strongly agreed that their skin felt relieved even after a single application, with an additional 43 (43%) agreeing. Regarding irritation, 40 (40%) participants reported that the formula did not irritate, while 30 (30%) agreed, 25 (25%) disagreed, and 5 (5%) strongly disagreed. In terms of stinging or burning sensations, 28 (28%) strongly agreed that the formula did not cause discomfort, 30 (30%) agreed, 32 (32%) disagreed, and 10 (10%) strongly disagreed. Participants' perceptions of skin nourishment varied, with 55 (55%) agreed that their skin felt better nourished, 19 (19%) strongly agreed, 19 (19%) disagreed, and 7 (7%) strongly disagreed. Ease of application was positively reported by 58 (58%) participants, with 29 (29%) strongly agreeing. Impressions of the formula's absorption into the skin were favorable, with 48 (48%) agreed, 36 (36%) strongly agreed, and 16 (16%) disagreed. Regarding skin greasiness, 39 (39%) agreed that the formula did not make their skin greasy, with 26 (26%) strongly agreed, 32 (32%) disagreed, and 3 (3%) strongly disagreed. Impressions of the sunblock product after first use varied, with 38 (38%) finding it very pleasant, 40 (40%) finding it pleasant, and 22 (22%) reporting an unpleasant experience (Table 2).

**Table 2:** Study participants' responses on day 1 after application.

Questions	Strongly agree	Agree	Disagree	Strongly disagree
My skin feels relieved even after a single application.	30 (30%)	43 (43%)	25 (25%)	2 (2%)
The formula does not irritate.	40 (40%)	30 (30%)	25 (25%)	5 (5%)
The formula does not cause stinging or burning.	28 (28%)	30 (30%)	32 (32%)	10 (10%)
My skin is better nourished even after the first application.	19 (19%)	55 (55%)	19 (19%)	7 (7%)
The formula is easy to apply.	29 (29%)	58 (58%)	10 (10%)	3 (3%)
This formula is quickly absorbed into the skin/does not leave white residues after.	36 (36%)	48 (48%)	16 (16%)	-
The formula does not make my skin greasy/oily after the first application.	26 (26%)	39 (39%)	32 (32%)	3 (3%)
What is the impression of the sunblock product after the first use?	Very pleasant	Pleasant	Unpleasant	Very unpleasant
	38 (38%)	40 (40%)	22 (22%)	-

On day 22 after application, participants' feedback regarding the SPF 100 sunblock revealed several insights. The majority found the formula easy to apply, with 68 (68%) agreed and 16 (16%) strongly agreed. Additionally, 58 (58%) agreed that their skin felt better moisturized after application, while 36 (36%) strongly agreed. A significant proportion, comprising 42 (42%) participants, reported that the formula did not irritate, with 32 (32%) agreeing. Similarly, 36 (36%) reported no stinging or burning sensations, with 29 (29%) agreed. In terms of sun protection, 50 (50%) agreed that the formula helped prevent facial redness provoked by the sun. However, 34 (34%) disagreed that it sufficiently protected their skin from the sun. Participants with rosacea noted that their symptoms did not worsen after product use, with 65 (65%) agreeing. Ease of incorporation into daily skin care routines was reported by 55 (55%) participants, with 36 (36%) strongly agreeing. Regarding absorption into the skin, 49 (49%) agreed that the formula was quickly absorbed, while 36 (36%) strongly agreed. Notably, 93 (93%) expressed willingness to buy the sunblock product. Additionally, 46 (46%) stated they would switch to the tested sunblock, citing better protection against skin redness (57, 57%) and minimal irritation (13, 13%) as primary reasons. An overwhelming majority, comprising 94 (94%) participants, expressed their intention to recommend the sunblock to family and friends (Table 3).

**Table 3:** Study participants' responses on day 22 after application.

Questions	Strongly agree	Agree	Disagree	Strongly disagree
The formula is easy to apply.	16 (16%)	68 (68%)	16 (16%)	-
My skin is better moisturized even after application.	36 (36%)	58 (58%)	3 (3%)	3 (3%)
The formula does not irritate.	32 (32%)	42 (42%)	23 (23%)	3 (3%)
The formula does not cause stinging or burning.	29 (29%)	36 (36%)	29 (29%)	6 (6%)
The formula helps to prevent facial redness provoked by the sun.	23 (23%)	50 (50%)	23 (23%)	4 (4%)
The formula sufficiently protects my skin from the sun.	33 (33%)	33 (33%)	34 (34%)	-
My rosacea symptoms did not get worse after using the product on my skin.	26 (26%)	65 (65%)	9 (9%)	-
The product is easy to incorporate into my daily skin care regimen.	36 (36%)	55 (55%)	7 (7%)	2 (2%)
The formula is quickly absorbed into the skin/does not leave white residues after application.	36 (36%)	49 (49%)	13 (13%)	2 (2%)
The formula does not make my skin greasy/oily after the first application.	19 (19%)	45 (45%)	36 (36%)	-
What is your overall impression of the sunblock product?	Very pleasant	Pleasant	Unpleasant	Very unpleasant
	13 (13%)	71 (71%)	16 (16%)	-

**Figure 1:**
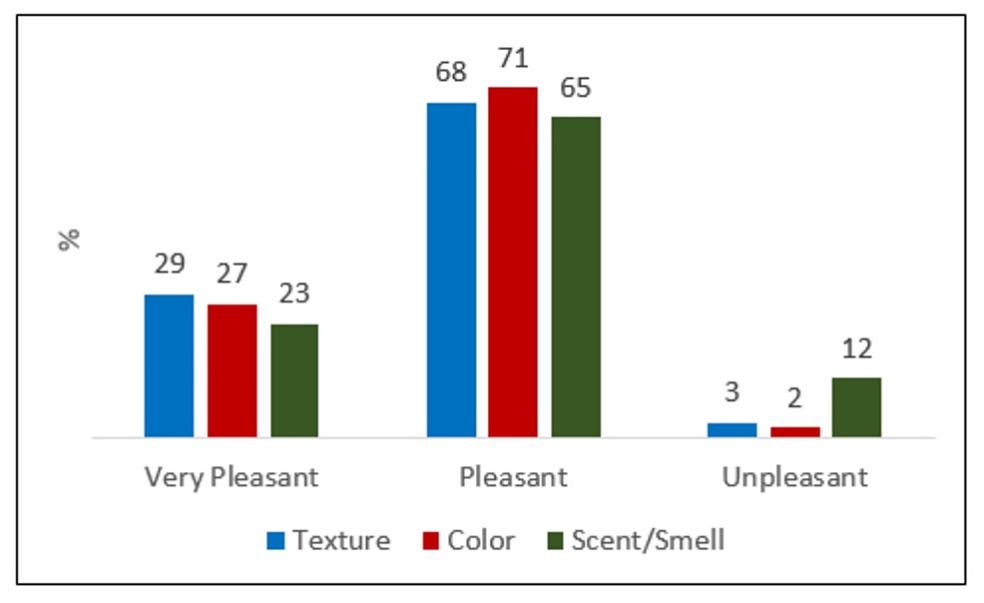
Responses of participants about texture, color, and smell characteristics of the product.

No adverse events were reported throughout the study duration, and there were no instances of study discontinuation. When queried about awareness of non-comedogenic sunblock options in Pakistan, 9 (9%) participants indicated awareness, while the majority (91, 91%) reported being unaware. Regarding satisfaction levels in prescribing Solero sunblock, a significant proportion of healthcare providers expressed satisfaction, with 76 (76%) being very satisfied and 20 (20%) being satisfied. Only a small percentage, comprising 4 (4%) respondents, reported being unsatisfied with prescribing Solero. The majority of participants rated the texture, color, and scent of the sunscreen product positively, with most finding them pleasant (texture, 68, 68%; color, 71, 71%; scent/smell, 65, 65%) or very pleasant (texture, 29, 29%; color, 27, 27%; scent/smell, 23, 23%) (Figure 1).

## Discussion

The classification of sunblocks in Australia into therapeutic goods or cosmetic sunblocks plays a significant role in understanding their intended purpose and formulation [19]. Cosmetic sunblocks, often referred to as secondary sunblocks, primarily focus on providing moisturization without significant sun protection, typically up to SPF 15. In contrast, primary sunblock formulations, with SPF values exceeding 15, are specifically designed to protect against UV rays [20-22].

The importance of sunblock in skin care routines is widely acknowledged by healthcare practitioners, as it effectively shields the skin from harmful UV radiation, thus preventing various adverse effects such as tanning, sunburns, and premature aging, and reducing the risk of skin cancer [22,23]. Guidelines from reputable organizations like the American Academy of Dermatology emphasize the necessity of sunblock with an SPF of 30 or above for individuals of all ages [24].

Age-specific recommendations for sunblock use further highlight its importance across different life stages. For instance, infants under six months are advised to avoid sunblock and instead rely on physical covering. Children aged six months to six years benefit from SPF 50 sunblock, while adolescents and adults are recommended SPF 50 or higher formulations. Specialized formulations, such as mineral sunblocks with SPF 40 or 50, cater to specific age groups, offering additional benefits like antioxidant protection [25].

Our study results suggest that SPF 100 sunblock is well-tolerated, effective, and acceptable among participants, with potential implications for its use in sun protection regimens and clinical practice. Research studies have demonstrated the efficacy of higher SPF sunblocks in providing enhanced protection against UV radiation. Studies by Liu et al. and Pissavini and Diffey revealed dose-response relationships, with higher SPF formulations showing exponential increases in protection [26-27]. Ou-Yang et al. highlighted the additional clinical benefits of SPF 70 or above sunblocks, suggesting that lower SPF formulations may not offer the same level of protection [13].

Our study contributes to this body of research by demonstrating the superiority of SPF 100 sunblock over SPF 50 sunblock in preventing sunburn and UV-induced erythema. Notably, 18% of our study participants used SPF 100 sunblock, indicating a preference for higher SPF formulations. Consistent with findings by Williams et al. and Kohli et al., our results emphasize the importance of using higher SPF sunblocks for optimal protection against sun damage [17-18].

Our study supports the recommendation for the use of SPF 100 sunblock, particularly in real-world conditions, where prolonged sun exposure occurs. By aligning with previous research and guidelines, our findings underscore the significance of sunblock in maintaining skin health and preventing sun-related skin conditions across all age groups.

Strengths and limitations

One of the strengths of this study is its prospective design, which allowed for the collection of real-world data on participants' preferences and experiences with SPF 100 sunblock (Solero). The inclusion of a diverse sample of participants with different age ranges and skin types enhances the generalizability of the findings. However, this study has some limitations. The relatively small sample size may limit the generalizability of the findings to larger populations. Additionally, the study duration was relatively short, and a longer-term follow-up would provide valuable insights into the durability of participant satisfaction and sunscreen efficacy over time. One potential source of bias was selection bias, as participants were recruited from a specific demographic range and geographic location, which may limit the generalizability of our findings to broader populations. Additionally, the voluntary nature of participation may introduce self-selection bias. To mitigate these biases, we employed standardized data collection procedures, ensured confidentiality and anonymity of responses, and utilized validated measurement tools. Despite these limitations, this study provides valuable insights into participants' experiences and satisfaction with SPF 100 sunblock in real-world settings.

## Conclusions

Our study indicates that SPF 100 sunblock (Solero) was well-received by the study participants, with positive perceptions of its effectiveness, ease of use, and safety. The majority of participants expressed satisfaction with the product and a willingness to continue using it, which may indicate a favorable safety profile and potential effectiveness in sun protection. While these findings are promising, larger and longer-term studies are warranted to substantiate its efficacy and suitability for broader use.
